# Binding of RPR260243 at the intracellular side of the hERG1 channel pore domain slows closure of the helix bundle crossing gate

**DOI:** 10.3389/fmolb.2023.1137368

**Published:** 2023-02-23

**Authors:** Eva-Maria Zangerl-Plessl, Wei Wu, Michael C. Sanguinetti, Anna Stary-Weinzinger

**Affiliations:** ^1^ Division of Pharmacology and Toxicology, University of Vienna, Vienna, Austria; ^2^ Department of Internal Medicine, Nora Eccles Harrison Cardiovascular Research & Training Institute, Division of Cardiovascular Medicine, University of Utah, Salt Lake City, UT, United States; ^3^ Department of Internal Medicine, Division of Cardiovascular Medicine, Nora Eccles Harrison Cardiovascular Research and Training Institute, University of Utah, Salt LakeCity, UT, United States

**Keywords:** human ether-à-go-go-related gene type 1, activators, deactivation gating, split channels, D540K, molecular modeling

## Abstract

The opening and closing of voltage-dependent potassium channels is dependent on a tight coupling between movement of the voltage sensing S4 segments and the activation gate. A specific interaction between intracellular amino- and carboxyl-termini is required for the characteristically slow rate of channel closure (deactivation) of hERG1 channels. Compounds that increase hERG1 channel currents represent a novel approach for prevention of arrhythmia associated with prolonged ventricular repolarization. RPR260243 (RPR), a quinoline oxo-propyl piperidine derivative, inhibits inactivation and dramatically slows the rate of hERG1 channel deactivation. Here we report that similar to its effect on wild-type channels, RPR greatly slows the deactivation rate of hERG1 channels missing their amino-termini, or of split channels lacking a covalent link between the voltage sensor domain and the pore domain. By contrast, RPR did not slow deactivation of C-terminal truncated hERG1 channels or D540K hERG1 mutant channels activated by hyperpolarization. Together, these findings indicate that ability of RPR to slow deactivation requires an intact C-terminus, does not slow deactivation by stabilizing an interaction involving the amino-terminus or require a covalent link between the voltage sensor and pore domains. All-atom molecular dynamics simulations using the cryo-EM structure of the hERG1 channel revealed that RPR binds to a pocket located at the intracellular ends of helices S5 and S6 of a single subunit. The slowing of channel deactivation by RPR may be mediated by disruption of normal S5-S6 interactions.

## Introduction

In the human heart, hERG1 (human *ether-à-go-go-*related gene type 1, Kv11.1) channels conduct the rapid delayed rectifier K^+^ current *I*_Kr_ (Sanguinetti et al., 1995; Trudeau et al., 1995) that contributes to action potential repolarization of cardiomyocytes. Several common medications that block hERG1 channels as an unwanted side effect cause prolongation of the QTc interval and an increased risk of arrhythmia (Fenichel et al., 2004). Compounds that enhance hERG1 currents (hERG1 activators) were more recently discovered and have been proposed as potential pharmacotherapy for congenital long QT syndrome (LQTS) and to counteract the effects of hERG1 blockers (Kang et al., 2005; Gerlach et al., 2010; Szabo et al., 2011; Sanguinetti, 2014; Mannikko et al., 2015). The mechanisms of action of hERG1 activators are diverse, but all affect channel gating by altering the kinetics and voltage dependence of activation, deactivation and/or inactivation. For example, RPR260243 (RPR or (3R, 4R)-4-[3-(6-methoxyquinolin-4-yl)-3-oxo-propyl]-1-[3-(2,3,5-trifluorophenyl)-prop-2-ynyl]-piperidine-3-carboxylic acid) dramatically slows the rate of deactivation (Kang et al., 2005) and induces a positive shift in the voltage dependence of inactivation of hERG1 channels (Perry et al., 2007). Interestingly, in zebra fish hearts with dofetilide-induced arrhythmia, RPR can restore a normal rhythm (Shi et al., 2020). Furthermore, recent electrophysiological studies have shown that RPR can rescue accelerated deactivation gating by a LQTS causing mutation (R56Q) in hERG1 (Kemp et al., 2021).

The properties of hERG1 channel gating have been extensively studied. Channels are opened (activated) and inactivated by membrane depolarization. In response to membrane repolarization, channels rapidly recovery from inactivation into an open state before they slowly close (deactivate). Ion permeation in voltage-gated K^+^ (Kv) channels, including hERG1, is controlled by an intracellular activation gate that is formed by a crossing of the α-helical S6 transmembrane segments that line the central cavity (Long et al., 2005a). Kv channels are in a non-conducting closed state when crossing of the S6 segments forms an aperture that is too narrow to allow passive flux of a hydrated K^+^ ion. In the canonical model of Kv channel gating, membrane depolarization induces an outward movement of the S4 transmembrane segments that is electromechanically coupled *via* the S4-S5 linkers to opening of the activation gate (i.e., outward splaying of the S6 segments) (Long et al., 2005b). This model does not fully apply to hERG1 channels because severing the covalent link between the voltage sensor domain (VSD) and the pore domain at the S4-S5 linker in hERG1 to form a “split hERG1 channel” has only minor effects on channel function (Lörinczi et al., 2015). Moreover, cryo-electron microscopy structures of the rat EAG1 and the transmembrane part of the human ERG channel revealed that the S4-S5 linker is a short loop with only 5 residues as opposed to the long 15 residue α-helix in the Kv1.2-2.1 channel, suggesting that it does not act as a mechanical lever to link movement of S4 segments to outward splaying of the S6 segments (Whicher and MacKinnon, 2016; Asai et al., 2021).

Herg1 channels are formed by two alternatively N-terminal spliced variants, hERG1a and hERG1b. Channels formed by coassembly of hERG1a subunits alone deactivate much slower than channels formed by hERG1b alone, and heteromultimeric channels have an intermediate rate of deactivation that more closely matches *I*_Kr_ recorded in native cardiomyocytes (London et al., 1997). We previously used a scanning mutagenesis approach to identify specific residues in hERG1a that might contribute to the RPR binding site. The interacting residues are located in the N-terminal end of the S5 segment that connects to the S4-S5 linker and nearby region of the C-terminal end of the S6 segment. The location of these key residues suggests that RPR may slow deactivation by interfering with electromechanical coupling between the VSD and the pore domain. The first goal of our study was to determine if RPR can slow deactivation of split hERG1a channels that lack a covalent link between the VSD and the pore domain.

Slow deactivation of wild-type (WT) hERG1a under normal conditions is dependent on the slow, voltage-dependent rate of VSD movement (Goodchild et al., 2015) and a structural interaction between intracellular N- and C-terminal domains of the channel subunits. The N-terminal structure (Cabral et al., 1998) includes a Per-Arnt-Sim (PAS, residues 26–135) domain and a PAS-cap (residues 1–26) domain that together are called the eag domain. Detailed study has revealed specific interaction between charged residues in the PAS-cap region of the amino terminus and the C-linker of the carboxyl terminus of an adjacent subunit of the homotetrameric hERG1a channel (Gustina and Trudeau, 2011; Gianulis et al., 2013; Li et al., 2014; Ng et al., 2014). Removal of the N-terminus precludes these interactions and markedly accelerates the rate of hERG1a channel deactivation (Schönherr and Heinemann, 1996; Cabral et al., 1998). The second goal of our study was to determine if the pronounced slowing of deactivation by RPR might result from stabilizing the interaction between the amino-terminus with another region of the hERG1a subunit.

Wild-type (WT) hERG1a channels are in a closed, non-conducting state at transmembrane potentials more negative than −60 mV. A charge-reversing amino acid substitution of Asp540, a residue located at the junction of the S4 segment and S4-S5 linker, dramatically disrupts normal channel gating. Similar to WT channels, D540K hERG1a channels are mostly closed at −70 mV and their open probability (P_o_) is progressively increased over the voltage range of −60–40 mV. In addition, D540K channels can also be induced to open in response to membrane hyperpolarization and P_o_ is progressively increased over the voltage range of −80–−140 mV (Sanguinetti and Xu, 1999; Mitcheson et al., 2000). The final goal of our study was to determine the effects of RPR on these two different modes of hERG1a channel gating.

## Materials and methods

### Molecular biology

WT human *ERG1* (*KCNH2*) isoform 1a cDNA (NCBI Reference sequence: NM_000238) and cDNA encoding an amino-terminal deleted (N-del) hERG1a subunit lacking residues 2–354 were inserted into the pSP64 oocyte expression vector (Sanguinetti et al., 1995; Spector et al., 1996). A partial carboxyl-terminal deleted (C-del) hERG1a, consisting of residues 1–698 linked by XhoI (CTCGAG) to residues 1,018–1,159, was constructed and inserted into the pSP64 vector as previously described (Wu and Sanguinetti, 2016). Separate VSD and pore domain modules of “split hERG1” were constructed as described by Lorinczi et al. (Lörinczi et al., 2015) with modifications described by Wu et al. (Wu et al., 2016). The two separate split hERG1a constructs in the pSP64T vector encoded residues 1–545 (VSD module) and residues 546–1,159 (pore module). All constructs were verified by DNA sequence analysis. To prepare cRNA for use in oocyte expression studies, plasmids were linearized with EcoRI prior to *in vitro* transcription using the mMessage mMachine SP6 kit (Ambion, Austin, TX).

### Isolation and injection of oocytes

Ovarian lobes from *Xenopus laevis* were harvested, treated with collagenase and oocytes harvested as previously described (Garg et al., 2012) using procedures approved by the University of Utah Institutional Animal Care and Use Committee. Isolated oocytes were incubated at 17°C in Barth’s solution. Individual oocytes were injected with 1 ng cRNA encoding WT channels and 5–10 ng encoding N-del or C-del hERG1a channels. For expression of split hERG1a channels, oocytes were co-injected with 12–20 ng each of the cRNAs encoding the VSD and pore modules.

### Voltage clamp data acquisition and analysis

Currents and transmembrane voltage were measured from single oocytes 1–4 days after cRNA injection using standard two-electrode voltage-clamp techniques (Stühmer and Parekh, 1995). Agarose cushion electrodes (Schreibmayer et al., 1994) were fabricated from glass micropipettes (TW100F-4, from World Precision Instruments, Inc., Sarasota, FL) and backfilled with 3 M KCl after their tips were plugged with a 3 M KCl/1.2% agarose solution.

A GeneClamp 500 amplifier, Digidata 1322 A data acquisition system, and pCLAMP 8.2 software (Molecular Devices, Inc., Sunnyvale, CA) were used for data acquisition and analysis of voltage clamp experiments. Origin (version 8.6) software (OriginLab Corp, Northampton, MA) was used for further data analysis and to prepare graphs and figures. Data are expressed as mean ± S.E.M. (*n* = number of oocytes). Where appropriate data were analyzed by two-way ANOVA or paired *t*-test. A *p*-value <0.05 was considered significant.

Concentration-response relationships for RPR effects on peak outward current during a depolarizing pulse (*I*_peak_), peak tail current (*I*_tail-peak_) and the time constant for deactivation (*τ*_deact_) of tail current (*I*_tail_) were fitted with a logistic equation:

$$y=A_{\max}+\frac{A_{\min}-A_{\max}}{1+{\left(\frac{\left[RPR\right]}{{EC}_{50}}\right)}^{n_{H}}}$$

where *A*_max_ and *A*_min_ are the maximum and minimum values of the measured parameter, EC_50_ is the concentration required for half maximal effect and *n*_H_ is the Hill coefficient.

Current-voltage (I-V) relationships were plotted after normalizing data relative to the peak outward current (*I*_max_) measured for each oocyte under control condtions. For most channel types, the voltage dependence of activation, or conductance-voltage (*G-V*) relationship was determined by plotting the normalized values of peak tail current (*I*_tail-peak_) as a function of *V*_t_ and fitting the resulting relationship with a Boltzmann function:

$$\frac{g}{g_{\max}}=\frac{1}{1+e^{\left(V_{0.5}-V_{t}\right)/k}}$$

where *V*_0.5_ is the half-voltage point and *k* is the slope factor of the relationship. The conductance-voltage (*G-V*) relationship for split hERG1a channels was determined as described previously (Wu and Sanguinetti, 2016) assuming the channel gating consists of four independent closed state transitions followed by a final concerted transition to the open state (Koren et al., 1990; Zagotta and Aldrich, 1990; Gonzalez et al., 2000):

$$C_{o}\overset{\frac{1}{4}K}{↔}\;C_{1}\overset{\frac{2}{3}K}{↔}\;C_{2}\overset{\frac{3}{2}K}{↔}\;C_{3}\overset{4K}{↔}\;C_{4}\overset{K_{o}}{↔}\;O$$

where *K = K(0)* exp (−*z*_*1*_ *FV*/*RT*) and *K*_*o*_ *= K*_*o*_ *(0)* exp (−*z*_*2*_ *FV*/*RT*). The normalized *G-V* relationship is described by:

$$\frac{g}{g_{\max}}={\left(1+K_{o}+4K_{o}K+6K_{o}K^{2}+4K_{o}K^{3}+K_{o}K^{4}\right)}^{-1}$$

Deactivation of hERG1 channel currents was determined by applying a prepulse to a fixed potential (0 or 40 mV), followed by repolarization to a return potential (*V*_ret_) that was varied in 10-mV increments and ranged from −140 to −40 mV. The rate of hERG1 channel current deactivation was determined by fitting the time-dependent decay of *I*_tail_ to monoexponential or biexponential functions:

$$I_{tail}\left(t\right)=Ae^{-t/\tau_{deact}}+C$$

$$I_{tail}\left(t\right)=A_{f}e^{-t/\tau_{f}}+A_{s}e^{-t/\tau_{s}}+C$$

where *τ*_deact_ is the time constant for monoexponential current deactivation, *τ*_f_ and *τ*_s_ are the fast and slow time constants of bi-exponential current deactivation, and *A*_s_ and *A*_f_ are the relative amplitude of the slow and fast components of deactivating tail current.

### Solutions and drugs

Isolated oocytes were stored in Barth’s solution that contained (in mM): 88 NaCl, 1 KCl, 0.41 CaCl_2_, 0.33 Ca(NO_3_)_2_, 1 MgSO_4_, 2.4 NaHCO_3_, 10 HEPES, 1 pyruvate, plus gentamycin (50 mg/L), amikacin (100 mg/L) and ciprofloxacin (25 mg/L); pH 7.4 with NaOH. For most of the voltage-clamp experiments, oocytes were bathed in a low K^+^ extracellular solution that contained (in mM): 96 mM NaCl, 2 mM KCl, 1 mM CaCl_2_, 1 mM MgCl_2,_ 5 mM HEPES; pH adjusted to 7.6 with NaOH. Currents conducted by split hERG1a channels were recorded from oocytes bathed in a high K^+^ extracellular solution that contained (in mM): 104 mM KCl, 1 mM CaCl_2_, 1 mM MgCl_2,_ 1 mM HEPES; pH adjusted to 7.6 with NaOH. RPR260243 (ChemShuttle, Wuxi, China) was dissolved in DMSO to make a 10 mM stock solution and stored at −20°C. Final RPR concentrations were obtained by dilution of the stock solution with extracellular saline solution.

### Molecular Modeling

Docking of RPR into the recently solved cryo-EM structure of hERG1 (open state, PDB: 5VA1, Wang and Mackinnon, 2017) was performed using the program Gold 4.0.1 (Cambridge DataCentre, Cambridge, United Kingdom) (Jones et al., 1995). To introduce protein flexibility, 20 snapshots, derived from previous WT hERG1 all-atom molecular dynamics simulations were used for docking (Zangerl-Plessl et al., 2020). Coordinates of the geometric center calculated among residues V549, L550, L553, F557, N658, I662, L666 and R681 (Perry et al., 2007; Gardner and Sanguinetti, 2015) were taken as binding site origin. The side chains of these residues were kept flexible. The binding site radius was set to 15 Å and 150,000 operations of the GOLD genetic algorithm were used to dock the compound.

## Results

### Split hERG1 channels

**FIGURE 1:**
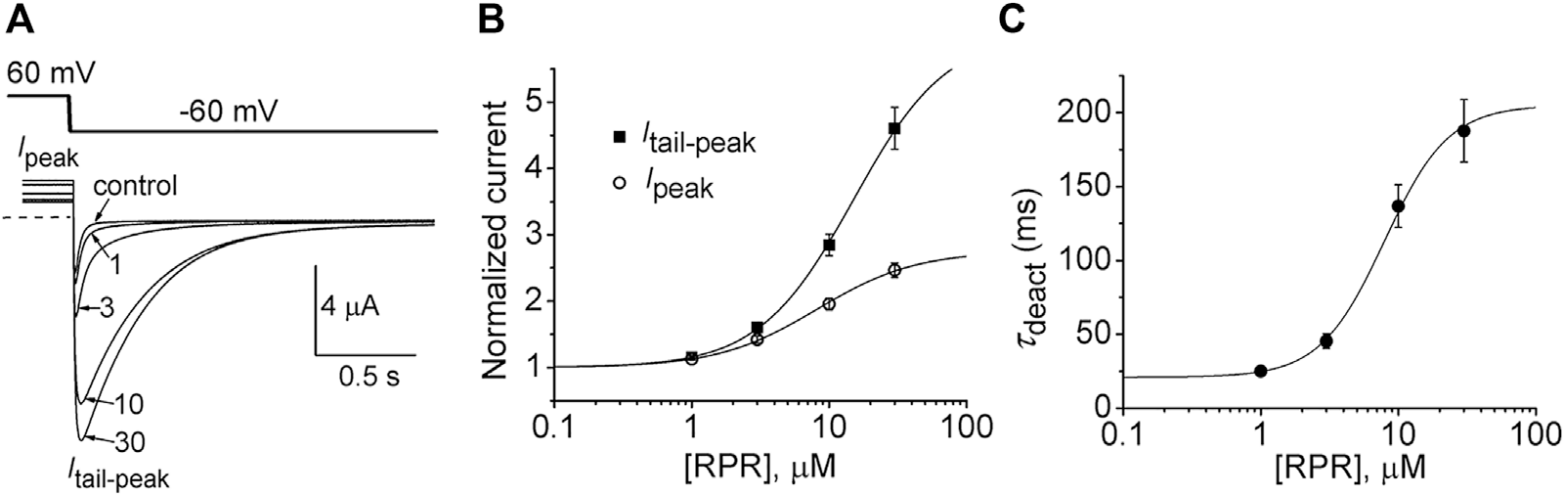
RPR activates split hERG1a channel currents. **(A)** Voltage clamp protocol and corresponding split hERG1a currents recorded from an oocyte before (control) and after treatment with indicated concentrations (in µM) of RPR. Oocyte was bathed in a solution containing 104 mM KCl. Dotted line indicates zero current level. **(B)** Concentration-response relationship for the effects of RPR on *I*_tail-peak_ (EC_50_ = 15.0 ± 1.9 µM, *n*_H_ = 1.3 ± 0.04, *n* = 7) and *I*_peak_ (EC_50_ = 8.2 ± 1.0 µM, *n*_H_ = 1.2 ± 0.06, *n* = 7). **(C)** Concentration-response relationship for the effects of RPR on *τ*_deact_ measured at −60 mV (EC_50_ = 7.9 ± 1.0 µM, *n*_H_ = 1.9 ± 0.1, *n* = 5).

In the orthodox structural model of Kv channel gating (Long et al., 2005b), intramembrane displacement of the VSD (specifically the S4 segments) in response to changes in transmembrane voltage is mechanically linked *via* the S4-S5 linkers to the opening and closing of the activation gate (the S6 bundle crossing). Contrary to the assumed structural requirements of this model, a recent study (Lörinczi et al., 2015) found that severing the covalent link between the VSD and the pore domain of KCNH family channels (EAG1, EAG2, hERG1) did not appreciably alter channel gating. Subunits were split apart at the S4-S5 linker into two separate constructs that when coexpressed in *Xenopus* oocytes resulted in assembly of functional channels that retained relatively normal biophysical properties (Lörinczi et al., 2015). We used the split hERG1a channel to test whether the slowing of deactivation induced by RPR was dependent on covalent linkage between the VSD and pore domains. Oocytes expressing split hERG1a channels were bathed in a high K^+^ (104 mM) solution and the effects of RPR (1–30 µM) were determined on currents elicited by repetitive pulsing to 60 mV from a holding potential of −70 mV (Figure 1A). The cumulative concentration response relationships for RPR on *I*_peak_ (EC_50_ = 8.2 ± 0.8 µM; *n* = 7) and *I*_tail-peak_ (EC_50_ = 15.0 ± 1.9 µM; *n* = 7) are plotted in Figure 1B. RPR slowed the rate of deactivation at a *V*_ret_ of −60 mV with an EC_50_ of 7.9 ± 1.0 µM (*n* = 5, Figure 1C). As previously reported, the EC_50_ for effects of RPR on *I*_tail-peak_ and *V*_0.5_ of deactivation for WT channels was 15 µM and 8 μM, respectively (Perry et al., 2007). Thus, the efficacy of RPR is similar for WT and split hERG1a channels.

**FIGURE 2:**
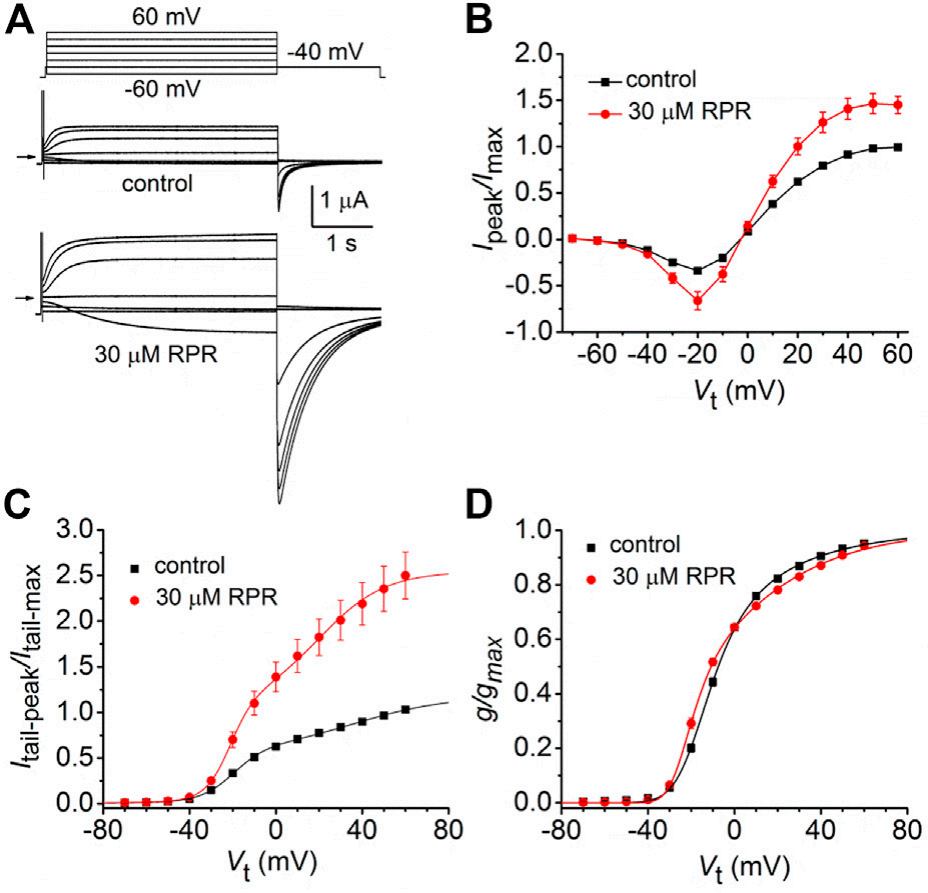
Effects of 30 µM RPR on split hERG1a channel currents. **(A)** Voltage clamp protocol and corresponding split hERG1a currents recorded from an oocyte before (control) and after treatment with 30 µM RPR. Oocyte was bathed in high K^+^ (104 mM) extracellular solution. Horizontal arrow indicates zero current level. **(B)** Effects of RPR on normalized *I*_*peak*_ *-V* relationship. Currents were increased by RPR (*n* = 7); *p* < 0.0001, 2 way ANOVA). **(C)** RPR increases the magnitude of *I*_tail-peak_ (*n* = 7; *p* < 0.0001, 2-way ANOVA). Currents were normalized relative to *I*_tail-max_ under control conditions. **(D)** *G-V* relationships. Averaged data from panel C were normalized relative to *I*_tail-max_ under each condition and fitted to a 6-state model of channel gating (smooth curves) as described in the Methods. Parameters for model under control conditions were: *z*_1_ = 1.71, *z*_2_ = 0.73, *K*(0) = 0.155, *K*_*o*_(0) = 0.32; in the presence of RPR: *z*_1_ = 2.98, *z*_2_ = 0.81, *K*(0) = 0.023, *K*_*o*_(0) = 0.513.

The effects of a high concentration (30 µM) of RPR on split hERG1a channel currents were examined in more detail. Currents were elicited with 4 s pulses applied to a *V*_t_ that was varied in 10-mV increments from −60 to 60 mV, and tail currents were measured at −40 mV (Figure 2A). RPR increased the magnitude of *I*_peak_ and especially *I*_tail-peak_. The averaged *I*_peak_ *-V*_t_ and *I*_tail-peak_-*V*_t_ relationships for multiple oocytes, normalized to the peak values under control conditions, are plotted in Figures 2B, C. Despite the large increase in the absolute values of *I*_tail-peak_, the shape of the *G-V* relationship describing the voltage dependence of activation was only slightly modified by 30 µM RPR (Figure 2D).

**FIGURE 3:**
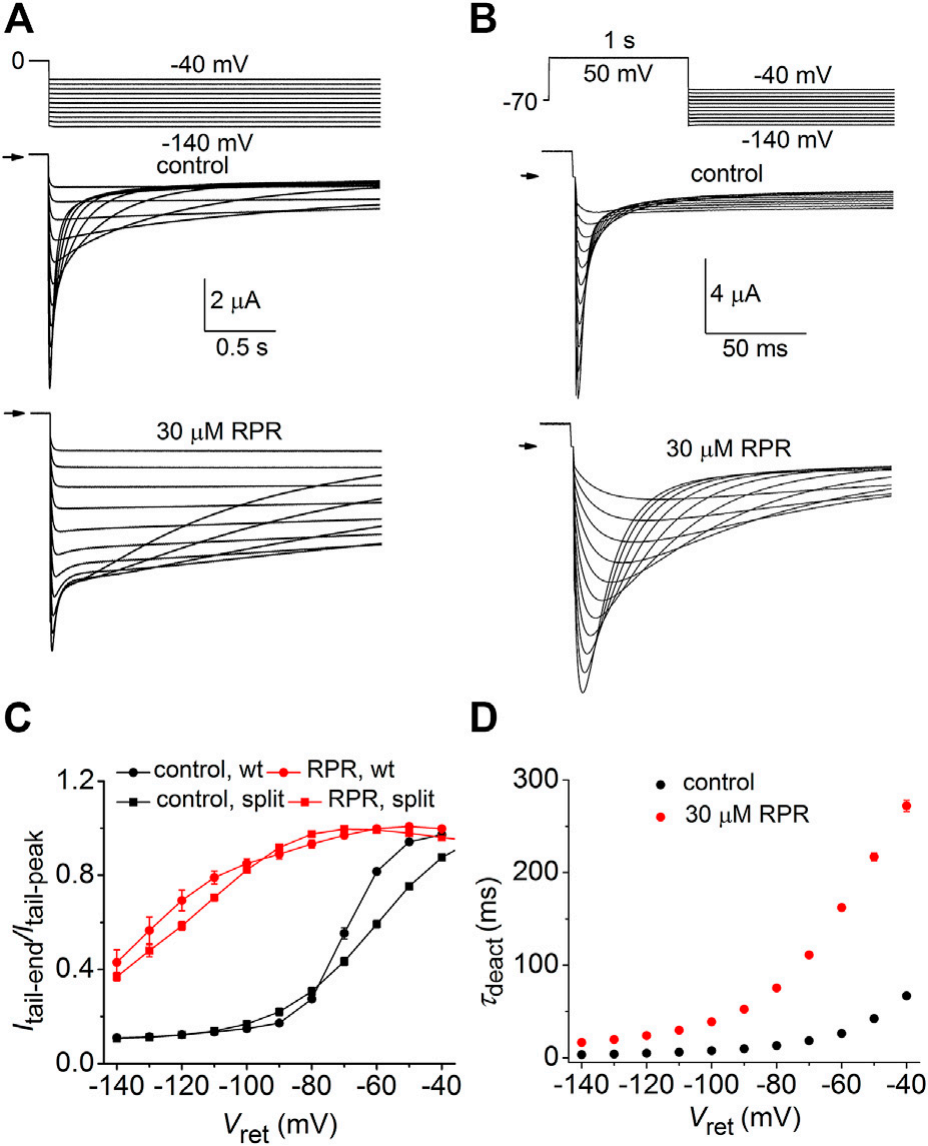
RPR slows deactivation rate of WT and split hERG1a channels. **(A)** Voltage pulse protocol and corresponding tail currents recorded from an oocyte expressing WT hERG1a channels before (control) and after treatment with 30 µM RPR (*n* = 5). From a holding potential of 0 mV, the cell was repolarized for 2.5 s to a variable *V*_ret_. Horizontal arrows indicate zero current level. **(B)** Voltage pulse protocol and corresponding tail currents recorded from a single oocyte expressing split hERG1a channels before (control) and after treatment with 30 µM RPR (*n* = 7). **(C)** Plot of *I*_tail-end_/*I*_tail-peak_ for WT channels (*n* = 5) and split hERG1a channels (*n* = 7) under control conditions and after treatment of oocytes with 30 µM RPR of split hERG1 channel (*n* = 7). *I*_tail-end_ was measured at 2.5 s for WT channel currents and at 25 m for the more rapidly deactivating split hERG1a channel currents. **(D)** RPR slowed *τ*_deact_ of split hERG1a channel currents (*n* = 7; *p* < 0.0001, 2-way ANOVA). For all experiments, oocytes were bathed in the high K^+^ (104 mM) extracellular solution.

We next determined the effects of RPR on deactivation of WT and split hERG1a currents. Channels were first fully activated by either setting the holding potential to 0 mV (WT channels, Figure 3A) or by pulsing to 50 mV for 1 s (split channels, Figure 3B). For both channel types, the membrane was then repolarized to a *V*_ret_ that was varied from −40 to −140 mV in 10-mV increments. Deactivation of WT channels is slow and biexponential, whereas split channel current is fast and mono-exponential. The different kinetic properties of WT and split channels make it difficult to directly compare the effects of RPR on the two channel types. Therefore, the slowing of deactivation of both WT and split hERG1a channel currents by RPR was quantified by plotting the ratio *I*_tail-end_/*I*_tail-peak_ as a function of *V*_ret_ (Figure 3C). For this analysis, *I*_tail-end_ was measured at 2.5 s for WT channel currents and at 25 m for the more rapidly deactivating split hERG1a channel currents. These times were chosen so that the ratio *I*_tail-end_/*I*_tail-peak_ was similar for both channel types under control conditions. RPR at 30 µM shifted the mid-point of the *I*_tail-end_/*I*_tail-peak_-*V*_ret_ relationships for both channel types by about −60 mV. RPR slowed deactivation of split channel currents by a factor of ∼4 (Figure 3D). Under control conditions *τ*_deact_ was 67 ± 3 m and 3.4 ± 0.1 m at a *V*_ret_ of −40–−140 mV, respectively. In the presence of 30 µM RPR,*τ*_deact_ was increased to 272 ± 6 m and 16.6 ± 0.7 m at −40 and −140 mV, respectively (*n* = 7). In summary, the relative slowing of deactivation rate by RPR was qualitatively similar for WT and split hERG1a channels. This finding indicates that slow deactivation induced by RPR is not dependent on a covalent linkage between the VSD and pore domain.

### N- or C-terminal deleted hERG1a channels

**FIGURE 4:**
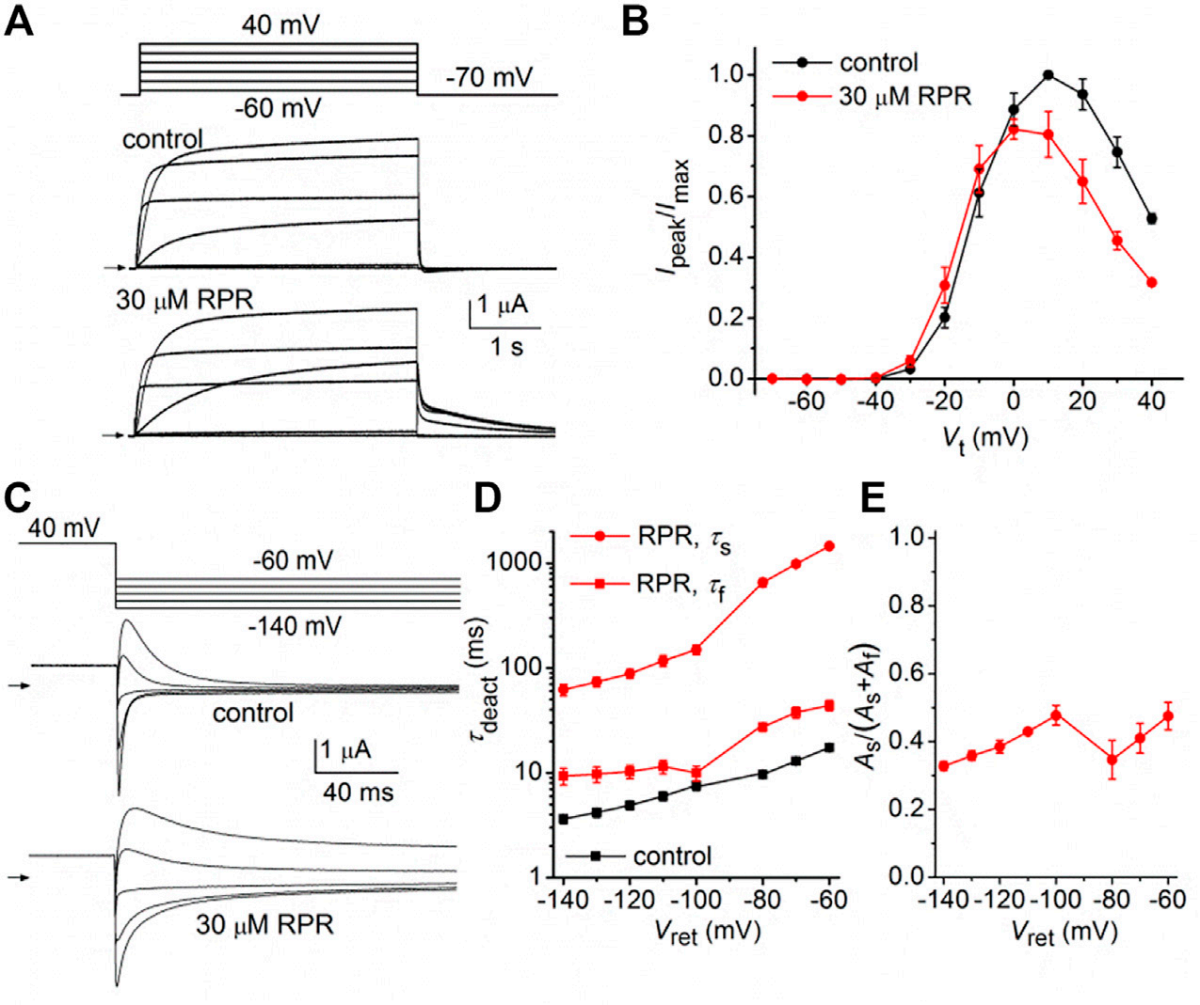
Effects of RPR on N-terminal deleted hERG1a channel currents. **(A)** Voltage pulse protocol and cooresponding N-del hERG1a channel currents recorded from an oocyte before (control) and after treatment with 30 µM RPR. Horizontal arrows indicate zero current level. **(B)** Effects of RPR on normalized *I*_*peak*_ *-V* relationship. RPR decreased *I*_*peak*_ at positive potentials (*n* = 5; *p* < 0.002, 2 way ANOVA). **(C)** Voltage pulse protocol and corresponding currents recorded before (control) and after treatment with 30 µM RPR. **(D)** Plot of *τ*_deact_ as a function of *V*_ret_. Time-dependent tail current decay was monoexponential under control conditions and bi-exponential in the presence of 30 µM RPR (*n* = 6). The fast rate of deactivation (τ_f_) measured after RPR was slower than τ_deact_ under control conditions (*p* < 0.001, 2-way ANOVA). **(E)** Relative amplitude of the slow component of deactivating tail current in the presence of 30 µM RPR (*n* = 6).

We next determined the effects of a high concentration (30 µM) of RPR on N-del hERG1a channels. In the representative experiment shown in Figure 4A, RPR slowed the rate of deactivation and diminished outward currents during step depolarizations to potentials >10 mV. The effect of RPR on current magnitude is summarized in Figure 4B where normalized *I*_peak_ is plotted as a function of *V*_t_ for multiple oocytes. The shape of the *I*_peak_-*V*_t_ was not altered by RPR, indicating that unlike WT channels (Perry et al., 2007), RPR does not inhibit inactivation of N-del channels. Deactivation of N-del hERG1a channels was characterized over a *V*_ret_ that ranged from −60 to −140 mV (Figure 4C). Deactivation of N-del hERG1 channels was rapid and reasonably fitted by a mono-exponential function. However, in the presence of RPR deactivation was much slower and bi-exponential (Figure 4D). Under control conditions *τ*_deact_ was 17.4 ± 0.6 m and 3.6 ± 0.3 m at −60–−140 mV, respectively. In the presence of 30 µM RPR, deactivation was biexponential and slower (*τ*_f_ = 44 ± 4.7 m; *τ*_s_ = 1,451 ± 63 m at −60 mV and *τ*_f_ = 9.3 ± 1.7 m; *τ*_s_ = 62 ± 7.7 m at −140 mV; *n* = 6). The relative contribution of the slow component of deactivation induced by RPR varied from 0.32 to 0.47 (Figure 4E). Thus, RPR does not require intact N-termini for it to slow deactivation of hERG1a channels.

**FIGURE 5:**
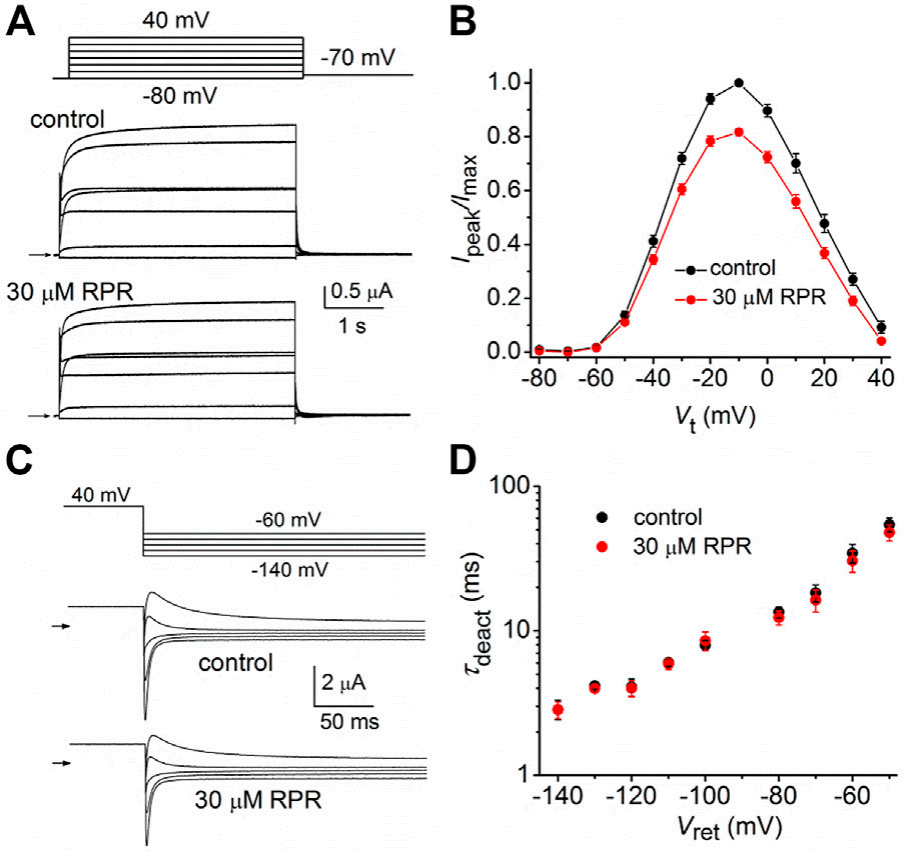
Effects of RPR on C-del hERG1a channel currents. **(A)** Voltage pulse protocol and corresponding C-del hERG1a channel currents recorded from an oocyte before (control) and after treatment with 30 µM RPR. Horizontal arrows indicate zero current level. **(B)** Averaged data for *I*_*peak*_ *-V* relationships. RPR decreased *I*_*peak*_ (*n* = 5; *p* < 0.001, 2 way ANOVA) **(C)** Voltage pulse protocol and corresponding tail currents recorded before (control) and after treatment with 30 µM RPR. Small horizontal arrows indicate zero current level. **(D)** RPR did not alter *τ*_deact_ (*n* = 5).

As reported previously (Wu et al., 2016), C-del hERG1a channels also deactivate rapidly. However, in contrast to N-del channels, 30 µM RPR had no effect on the kinetics of C-del channel currents (Figure 5A). RPR reduced the magnitude of outward currents by (e.g., by 18% ± 1% at −10 mV (*p* < 0.001), but did not alter the shape of the *I-V* relationship (Figure 5B) or *τ*_deact_ over a 100 mV range of *V*_ret_ (Figures 5C, D). Thus, deletion of a major region of the C-terminus prevents RPR-induced slowing of hERG1a channel deactivation. The mechanism of the RPR-induced reduction in outward currents observed at positive test potentials for both N-del and C-del channels was not investigated, but may result from channel block at the high concentration (30 μM) of the compound.

### D540K hERG1a channels

**FIGURE 6:**
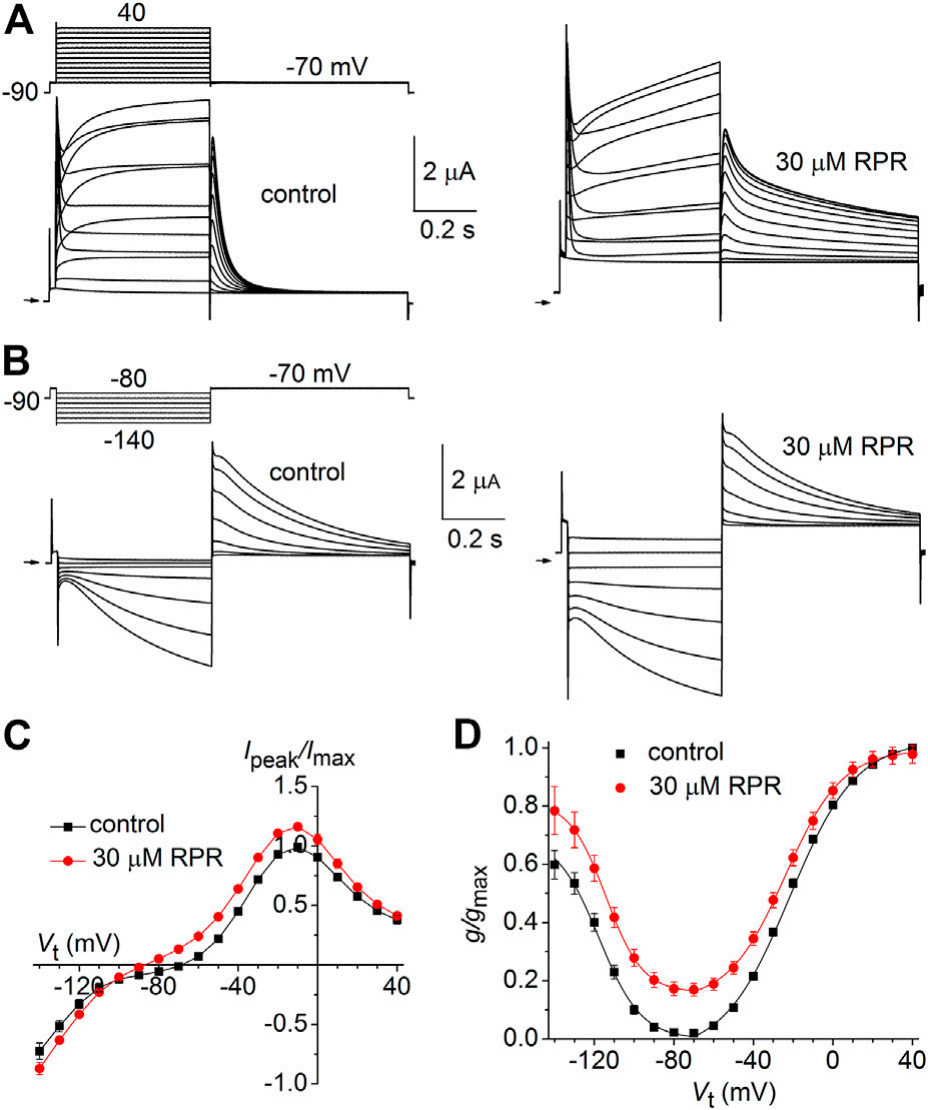
Effects of RPR on D540K hERG1a channel currents. **(A)** Voltage clamp protocol and corresponding currents recorded at a *V*_t_ ranging from −70 mV to 40 mV from an oocyte before (control) and after treatment with 30 µM RPR. Small horizontal arrows indicate zero current level. **(B)** Voltage clamp protocol and corresponding currents recorded at a *V*_t_ ranging from −80 mV to −140 mV from a single oocyte before (control) and after treatment with 30 µM RPR. **(C)** *I-V* relationships for 0.5 s test pulses (*n* = 10; *p* < 0.001, 2-way ANOVA). **(D)** Effect of RPR on the *G*-*V* relationship (*n* = 10). For control, *V*_0.5_ = −118 ± 3 mV (*k* = 10.6 ± 1.1) and −24.7 ± 4 mV (*k* = 7.2 ± 1.0). For 30 µM RPR, *V*_0.5_ = −114 ± 0.5 mV (*k* = 9.2 ± 0.3) and −23.9 ± 0.4 mV (*k* = 14.2 ± 0.4)).

Similar to other Kv channels, WT hERG1 channels are activated in response to membrane depolarization and are stabilized in a closed state at negative transmembrane potentials. This normal mode of gating is disrupted by a charge reversing mutation of Asp540 located at the C-terminal end of the S4 segment (Sanguinetti and Xu, 1999). D540K channels open in response to depolarization over a range of voltage that is similar to WT channels, but deactivate more rapidly upon repolarization of the membrane. In addition, D540K channels can also be opened in response to membrane hyperpolarization, reminiscent of a gating mode exemplified by the HCN family of pacemaker channels (Ludwig et al., 1999). The effects of RPR on D540K hERG1a deactivation might depend on the mode of channel gating, specifically whether channels are activated by depolarization or hyperpolarization. To examine this possibility, oocytes expressing these mutant channels were voltage clamped to a holding potential of −90 mV and currents were measured in response to a *V*_t_ that was varied from 40 mV to −140 mV. Each test pulse was preceded by a brief prepulse to −70 mV and tail currents were measured at −70 mV following each test pulse. To facilitate viewing the differential response to depolarizing *versus* hyperpolarizing pulses, currents for a single oocyte are plotted in two separate panels (A and B) in Figure 6. Currents elicited by depolarizations (to a *V*_t_ of −70–40 mV) activated and deactivated rapidly under control conditions (Figure 6A, left panel). RPR (30 µM) slowed both the rate of activation and deactivation in response to the same potential range of test pulses (Figure 6A, right panel). However, the rate of onset of currents activated by hyperpolarization (to a *V*_t_ of −80–−140 mV) and the rate of deactivation induced by return to the holding potential of −70 mV were unaffected by RPR (Figure 6B). The effect of RPR on the *I-V* relationship, normalized to the peak outward current recorded at −10 mV under control conditions, is plotted in Figure 6C. The reversal potential (*E*_rev_) of the *I-V* relationship was shifted to more negative potentials by RPR. *E*_rev_ is determined by the relative contribution of unsubtracted native inward leak current to the outward current conducted by heterologously expressed D540K hERG1a channels. In the presence of RPR, hERG1 channels remain open near *E*_rev_ (as indicated by increased slope conductance), offsetting the leak conductance more than under control conditions. To determine the effect of RPR on relative channel conductance over a broad range of voltage, *I*_*tail*_-peak was measured in response to both depolarizing and hyperpolarizing test pulses following a 1 s pulse to 40 mV to fully activate the channels. Normalized values of *I*_*tail*_-peak were plotted as a function of *V*_t_. The resulting averaged *G-V* relationships determined before and after 30 µM RPR were fitted with the sum of two Boltzmann functions (Figure 6D). RPR increased the minimum value of *g*/*g*_max_ (at *V*_t_ = −70 mV) from 0.02 ± 0.01 to 0.17 ± 0.02 (*n* = 10, *p* < 0.0001), reduced the slope factor for depolarization-activated currents, but did not appreciably alter the *V*_0.5_ values for channel activation in response to either depolarization or hyperpolarization.

**FIGURE 7:**
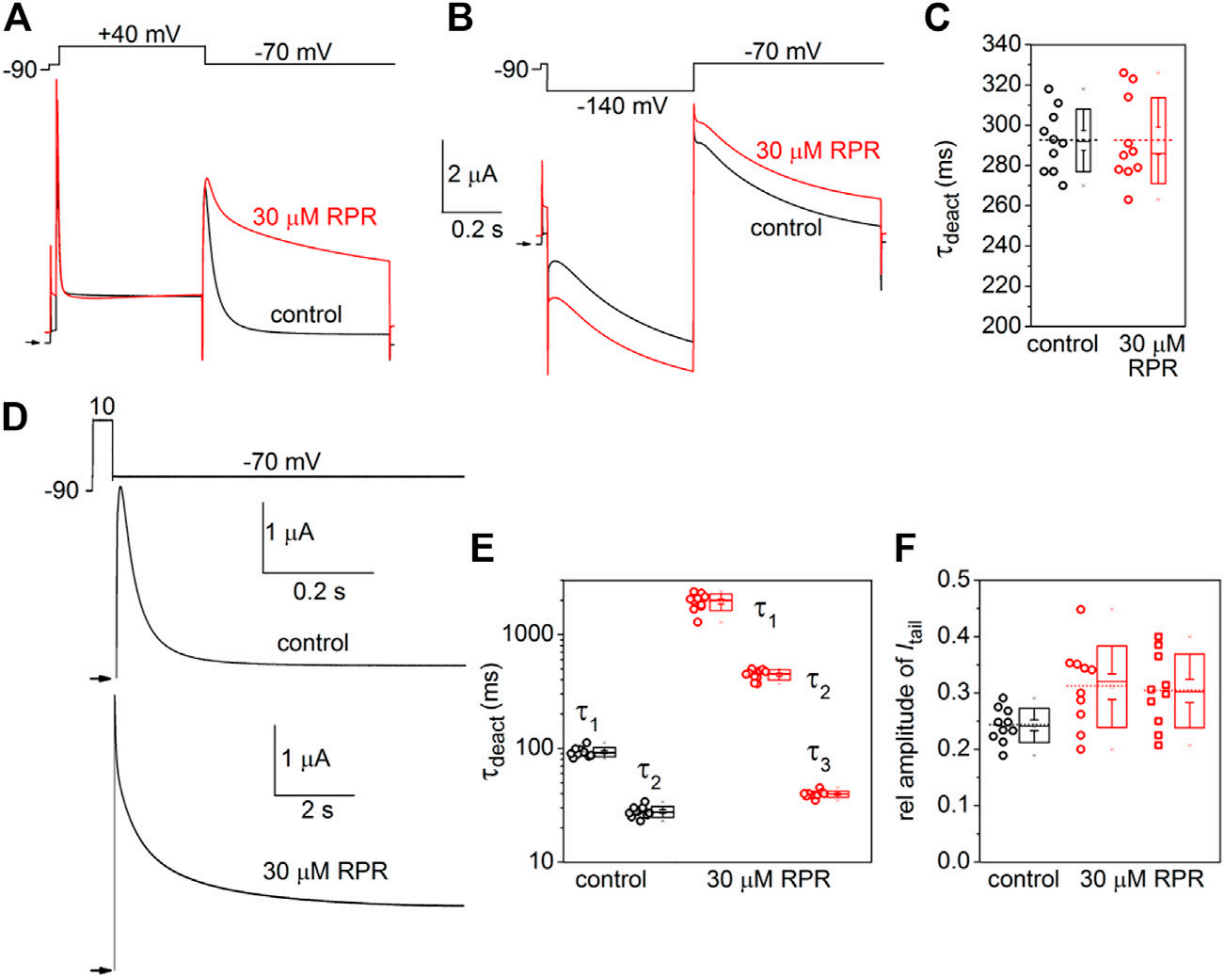
Differential effects of RPR on deactivation of D540K hERG1a channels opened by depolarizing or hyperpolarizing pulses. **(A)** RPR slowed the rate of current measured at a *V*_ret_ of −70 mV after channels were activated by a 0.5 s depolarization to 40 mV. **(B)** RPR did not alter the rate of current deactivation of current measured at a *V*_ret_ of −70 mV after channels were activated by a 0.5 s hyperpolarization to −140 mV. **(C)** Scatter plot for *τ*_deact_ measured at −70 mV following a 0.5 s pulse to −140 mV (*n* = 10). Plot to right of data indicates mean (dashed line), S.E.M. (whiskers) and S.D. (box). **(D)** Voltage pulse protocol and corresponding tail currents measured from an oocyte before (control) and after RPR. Channels were activated by a 0.5 s prepulse to 10 mV. Note difference in time scale for different conditions. **(E)** Scatter box plots for effect of 30 µM RPR on *τ*_deact_ measured at −70 mV following a 0.5 s pulse to 10 mV (*n* = 10). **(F)** Scatter box plots for relative amplitudes of *I*_tail_ components at −70 mV following a 0.5 s pulse to 40 mV (*n* = 10). For control: data represent A_1_/(A_1_+A_2_); for RPR, circles represent A_1_/(A_1_+A_2_+A_3_) and squares represent A_2_/(A_1_+A_2_+A_3_).

The effects of RPR on the kinetics of D540K hERG1a channel deactivation can be more easily seen when current traces are superimposed (Figures 7A, B). *I*_tail_ measured at −70 mV was slowed dramatically following a test pulse to 40 mV, but not after the pulse to −140 mV. The time-dependent decay of *I*_tail_ elicited after an activating pulse to −140 mV were best fit with a single exponential function and RPR had no effect on *τ*_deact_ (Figure 7C). *I*_tail_ elicited after an activating pulse to 40 mV was very slow in the presence of RPR. Therefore, tail currents were measured over a longer time period (Figure 7D). For this experiment, a 0.5 s pulse to 10 mV was followed by a 0.6 s (control) or 9 s (RPR) return pulse to −70 mV. For the experiment shown in Figure 7D, deactivation of *I*_tail_ was rapid and best fit with a two exponential function under control conditions (*τ*_f_ = 24 m, *τ*_s_ = 79 m). In the presence of 30 µM RPR, tail currents deactivated much slower and adequate fitting required a three exponential function (*τ*_1_ = 1.97 s, *τ*_2_ = 442 m, *τ*_3_ = 35 m). The analysis of deactivation using this pulse protocol was conducted for 10 oocytes and the average results for the multiple time constants and relative amplitudes (A_x_/A_total_) are plotted in Figures 7E, F. In summary, although RPR slows the rate of closure of channels opened by depolarization, it did not alter the rate of deactivation of channels opened by hyperpolarization.

### Docking of RPR to the full length WT hERG1 channel in open conformation

**FIGURE 8:**
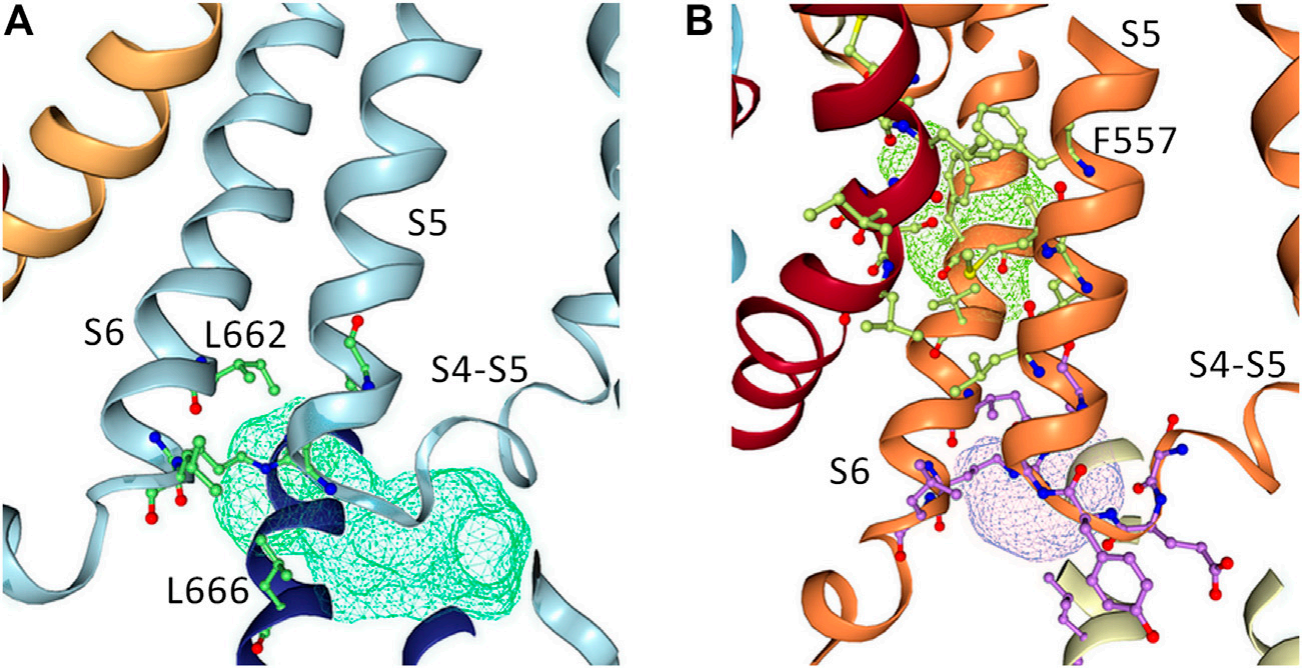
Pocket analysis of the RPR binding site in EAG1 and the hERG1 channel. **(A)** Pocket analysis in the hERG1 cryo EM structure (pdb: 5VA1). Shown in blue are the S4-S5 linker, the S5 as well as the S6 helices. Pocket-restricting residues are represented in green. The light blue mesh indicated the pocket found by the program DoGSiteScorer. **(B)** Pocket analysis of a MD snapshot of the hERG1 channel. Shown in orange are the S4-S5 linker, the S5 as well as the S6 helices. The light purple mesh indicated the pocket found by the program DoGSiteScorer. Pocket-lining residues are represented in green and purple.

**FIGURE 9:**
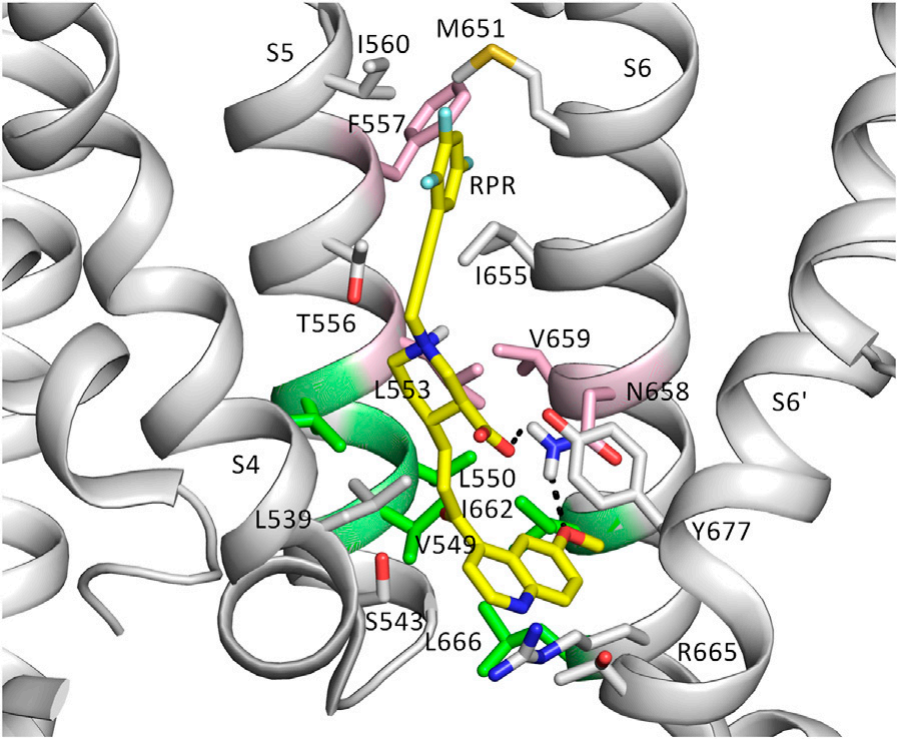
The putative binding site of RPR. Binding residues and RPR are presented as sticks. RPR is shown in yellow. Residues represented in pink are known from experiments to prevent slowing of deactivation and reduced the shift of V_0.5_ of inactivation by RPR. Those in green weakened the effect of deactivation, but did not change the effect of RPR an inactivation. Gray residues are within 6 Å of RPR.

**FIGURE 10:**
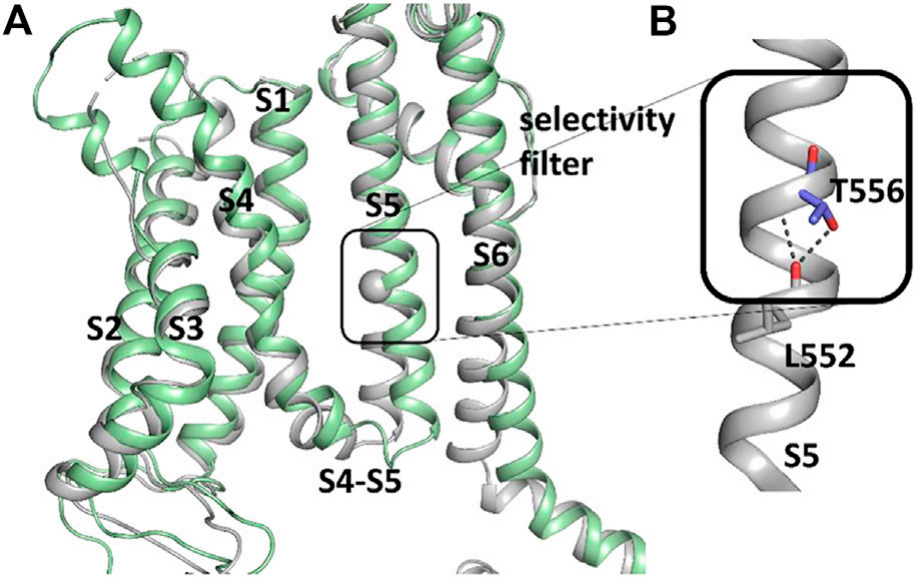
Difference between hERG1 and rEAG1 structure and T556 interactions. **(A)** Cryo-EM structure of the membrane-embedded part of one subunit of the hERG1 channel (pdb: 5VA1) represented in gray and the EAG1 structure (pdb: 5K7L) in green. The c-alpha atom of T556 is represented as sphere. **(B)** Part of the S5 of the hERG1 channel structure with details of the T556 h-bond formation to the backbone of L552. T556 and L552 are shown as sticks.

To further investigate how RPR might affect hERG1 channel gating, docking into MD derived snapshots of the recent cryo-EM structure in the open state (pdb: 5va1), with the voltage sensor in the “up-state,” was performed. While all residues identified previously as important for RPR interaction *via* Ala-scan mutagenesis (Perry et al., 2007) are in close proximity in the 3D coordinates, there is very little space between the interacting residues for RPR to bind in the cryo-EM structure “snapshot”. The lack of a sufficiently large and drug accessible pocket is shown in Figure 8A. Pockets were calculated using the protein pocket predicting program (Schöning-Stierand et al., 2020; Fährrolfes et al., 2017) (https://proteins.plus/, 2022; Volkamer et al., 2012). Given the highly dynamic nature of the hERG1 channel, and in particular of its aromatic side chains in the S5/S6 region (e.g., (Knape et al., 2011; Perissinotti et al., 2019)), drug accessible pockets in MD snapshots were analyzed. Indeed, in many snapshots relatively minor changes in backbone and side-chain orientations (Supplementary Figure S1), led to suitable drug accessible pockets in the region previously suggested as the RPR binding site (Figure 8B). Ensemble docking, using snapshots from previous WT MD simulations (Zangerl-Plessl et al., 2020) was performed. Randomly picked snapshots from MD were analyzed with DoGSiteScores, and conformations with suitable drug accessible pockets in the intracellular region between helices S5 and S6, were used for docking. Figure 8B shows a representative drug pocket from a MD snapshot. Due to conformational changes in helix S6 an accessible drug binding pocket could be formed, rendering residues up to F557 in the interface between S5 and S6 accessible. A representative docking pose of RPR is shown in Figure 9. In this docking, all residues identified previously by mutagenesis (Perry et al., 2007), are suggested to directly interact with the RPR molecule. While N658 is predicted to form two hydrogen bonds to the carboxy group and the metoxy quinoline ring of the drug, respectively, the majority of interactions are hydrophobic in nature. Residue R681, located in the C-linker, previously shown to account for differential sensitivities of ERG1 and ERG2 K+ channels for RPR (Gardner and Sanguinetti, 2015), is ∼10 Å away from the other binding residues and does not directly interact with RPR in this docking that is consistent with previously identified drug interaction residues residue. In addition, the binding site includes several weak hydrophobic contacts to 4 residues that were not identified as critical in the previous Ala-scan, as shown in Figure 9. Structural comparison of the open state structure with a closed homology model (based on Kv10.1, Figure 10A) reveals subtle structural rearrangements at the helix bundle crossing gate in the order of 2-3 Å. Importantly, these conformational changes involve the majority of residues, located in the putative RPR binding site (Perry and Sanguinetti, 2008). Bending of the lower part of helix S5 in the open state is stabilized *via* an intrahelical h-bond of T556 with the main chain carbonyl oxygen of residue L552, shown in Figure 10B. Interestingly, T556, which is replaced by an I558 in rat ERG3 channels, explains the reduced sensitivity towards RPR in these channels. Moreover, mutation of L552 A to Ala greatly enhanced the ability of RPR to slow hERG1a deactivation, far more than any other mutation investigated (Perry et al., 2007). Together, these findings suggest a potentially key role for the T556-L552 interaction in determining the relative activity of RPR. Docking predicts that RPR binds at the intracellular half between helices S5 and S6, thereby disrupting the extensive interaction interface between these chains. It is plausible that this disruption delays closure of the gate.

## Discussion

### hERG1 channel activators

Loss of function mutations in hERG1 are a common cause of LQTS (Curran et al., 1995), a disorder of ventricular repolarization that is associated with potentially life threatening arrhythmia and ventricular fibrillation (Jervell and Lange-Nielsen, 1957; Schwartz et al., 1991). The link between inhibition of *I*_Kr_ by common medications and QT interval prolongation, increased risk of torsades de pointes and sudden death in susceptible individuals prompted routine safety assessment of compounds during early stages of drug development for their propensity to block hERG1 channels. These efforts unexpectedly led to the discovery of several structurally diverse hERG1 activators, including RPR (Kang et al., 2005), PD-118057 (Zhou et al., 2005), NS1643 (Hansen et al., 2006), mallotoxin (Zeng et al., 2006), A-935142 (Su et al., 2009), ICA-105574 (Gerlach et al., 2010), KB130015 (Gessner et al., 2010), ginsenoside Rg3 (Choi et al., 2011), AZSMO-23 (Mannikko et al., 2015) and compounds related to NS1643 (Guo et al., 2014; Giacomini et al., 2015). Based on their ability to hasten cardiac repolarization and shorten QT interval, hERG1 activators represent a novel approach for the treatment of LQTS. The abundance of hERG1 activators is low compared to compounds that inhibit channel activity. In a survey of more than 60,000 compounds using concentrations of 30 or 100 μM, hERG activation was rare (<0.1%) when compared to hERG1 inhibition (84%) (Mannikko et al., 2015). Compounds activate hERG1 channel activity by a variety of mechanisms, including slowed deactivation, reduced inactivation, increased open channel probability, shift of activation to more negative potentials or a combination of two or more of these effects (Sanguinetti, 2014; Wu et al., 2016).

### Split hERG1 channels

The first hERG1 activator to be described in the literature was RPR, a compound that dramatically slows the rate of channel deactivation (Kang, et al., 2005). We previously used a site-directed mutagenesis approach to locate the putative RPR binding site on hERG1a channels (Perry, et al., 2007) and to explore the molecular basis for the differential sensitivity of ERG1, ERG2 and ERG3 channels to this compound (Perry and Sanguinetti, 2008; Gardner and Sanguinetti, 2015). In the present study we further investigated the mechanism of action of RPR by determining its effects on hERG1a channels lacking a covalent link between the VSD and the pore domain. Splitting hERG1a into two domains only modestly affected the gating properties of channels that were reconstituted after heterologous expression of the separate proteins in oocytes (Lorinczi, et al., 2015). Split channels retain their sensitivity to the gating modifiers ginsenoside Rg3 and RPR. Ginsenoside Rg3, a steroid glycoside shifts the voltage dependence of activation of split hERG1a channels by −14 mV, similar to its effects on WT channels (Wu, et al., 2016). In addition, *I*_tail-peak_ of split hERG1a channels was augmented much more by RPR (2.5-fold increase) and ginsenoside Rg3 (5-fold increase) compared to the ∼1.4-fold increase of WT channels by both compounds. The mechanism responsible for the greater effects of Rg3 and RPR on the magnitude of split channel current is unknown, but an increase in single channel Po seems most likely for Rg3 because it does not alter the voltage dependence of inactivation (Wu, et al., 2016). The slowed rate of channel closure induced by RPR is not caused by a slowed rate of intramembrane gating charge displacement in response to membrane repolarization. Even at a concentration of 30 μM, RPR did not alter the kinetics or magnitude of *Ig*_OFF_, the membrane repolarization-induced gating current that results from inward movement of the S4 segments (Abbruzzese, et al., 2010). In summary, RPR dissociates the normal coupling between the VSD and the S6 activation gate and slowed channel deactivation by this compound does not require a covalent link between the VSD and pore domains. This is perhaps not surprising since the biophysical properties of the split channel indicate that non-covalent interactions between the VSD and pore domain are sufficient to permit near normal gating.

### Role of cytoplasmic termini

The ability of RPR to alter channel gating was also assessed after truncation of the cytoplasmic N-terminus or deletion of the majority of the C-terminus of the hERG1a subunit. The unusually slow deactivation of WT hERG1a channels requires a structural interaction between the N- and the C-termini of a neighboring hERG1a subunit (Gustina and Trudeau, 2011; Gianulis et al., 2013; Li et al., 2014). Interaction between the N-terminus and the S4-S5 linker has also been proposed to modify the kinetics of channel gating (Li et al., 2010; de la Peña et al., 2011; Ng et al., 2011). More recently, specific charge-pair interactions between Arg56 (PAS domain) and Asp803 (cyclic nucleotide binding homology domain, CNBHD), plus Arg5 (PAS-cap) and Glu698/Glu699 (C-linker) were proposed to mediate the link between the N- and C-termini (Ng et al., 2014). The cryo-EM structure of the hERG1 channel in the open conformation supports these interactions (Wang and MacKinnon, 2017). Removal of the N-terminus of hERG1 greatly accelerates the rate of channel deactivation (Schönherr and Heinemann, 1996; Cabral et al., 1998). However, we found that RPR was still capable of slowing the rate of deactivation of N-del hERG1a channels. This finding rules out the possibility that the mechanism of RPR action is dependent on stabilization of specific interactions between an N-terminus and any other structural component of the hERG1a subunit.

The carboxyl-terminus of hERG1 subunits includes a cyclic nucleotide-binding homology domain (CNBHD) that is coupled to the S6 α-helical transmembrane segment by the C-linker (Brelidze et al., 2013). We found that C-del hERG1a channels were insensitive to the gating effects of RPR. This was not unexpected given our previous findings that specific residues within the C-linker could account for the differential sensitivity of ERG channels to the gating effects of RPR. The residues that comprise the putative binding pocket for RPR (Perry et al., 2007) are fully conserved between rat *ether-à-go-go*-related gene type 2 (rERG2) and hERG1, yet rERG2 channels are relatively insensitive to RPR. However, the cytoplasmic C-linker, composed of three α-helical domains differs by only 5 residues in rERG2 compared to hERG1. Mutation of these 5 residues in hERG1a to match those in rERG2 renders the channel almost insensitive to RPR, while the converse residue swapping makes rERG2 highly sensitive to RPR (Gardner and Sanguinetti, 2015). It is unknown whether these residues represent additional molecular determinants of RPR binding affinity, or if they contribute to RPR efficacy by an allosteric effect. Molecular modeling presented here supports an allosteric role rather than a direct binding, given the fact that these 5 residues are too distant (>10 Å, away) from the binding site consistent with extensive Ala-scanning (Perry et al., 2007).

The reduced sensitivity of rat ERG3 channels to RPR can be attributed to a single homologous residue in the S5 segment (Ile558 in rat ERG3, Thr556 in hERG1a) that is located in the putative RPR binding site (Perry and Sanguinetti, 2008). Molecular docking investigations based on the recent cryo-EM structure of the hERG1 channel in open conformation supports a binding site of RPR located at the intracellular ends of helices S5 and S6 and suggests that drug binding might delay closure of the pore module. Comparison of hERG channels with EAG1 channels, obtained with different voltage sensor conformations (Wang and MacKinnon, 2017; Mandala and MacKinnon, 2022) led to the proposal that voltage sensors transmit their force *via* the S5-S6 interface, the proposed binding region of RPR. It is thus plausible that binding of RPR considerably delays gate closure and thus deactivation, *via* hindering compression of the S5-S6 interface by the S4 helix.

RPR slows deactivation of D540K hERG1a channels that are opened by depolarization, but not by hyperpolarization of the membrane. To elucidate, why RPR does not alter deactivation of hyperpolarization-dependent channel opening, structural information about the D540K induced hyperpolarized open state would be necessary. Structures of the hyperpolarization-activated HCN1 channels (Lee and MacKinnon, 2017) suggest that the voltage sensors might move further inward upon hyperpolarization; however it is not clear if the D540K mutant would use the same principles. An important difference between hERG1 channels and HCN1 channels is the unusual length of the S4 helix in the latter, which contains two additional helical turns on the cytoplasmic side, a fact that has been described crucial for the unique gating (Lee and MacKinnon, 2017). Thus, it is currently unclear, if for example repositioning of the VSD causes unbinding of RPR, or RPR remains bound but no longer interferes with narrowing of the S6 bundle crossing. Future investigations, including extensive MD simulations on the D540K mutant channel might help shed light on this very interesting question.

hERG1 activators that are structurally unrelated to RPR can enhance outward currents by mechanisms other than attenuated deactivation. Examples include strong inhibition of inactivation by ICA-105574 (Gerlach et al., 2010. Mol Pharmacol vol 77; *p* 58–68), or increasing channel open probability by PD-118057 (Perry et al., 2007. PNAS vol 106, *p* 20,075–20080). Mutagenesis and docking studies suggest that these other two compounds bind to a hydrophobic pocket formed between the pore modules (S5/S6) of two adjacent subunits (Perry et al., 2007. PNAS vol 106, *p* 20,075–20080; Garg et al., 2012. Mol Pharmacol vol 83, *p* 805–813) rather than the S5/S6 regions of a single subunit as reported here and suggested previously (Perry et al., 2007) for RPR. Given the homotetrameric structure of hERG1a channels, a single channel should contain four equivalent binding sites for each activator, raising the possibility that agonist effects might be accumulative in accordance with the accessibility of these multiple sites. This prediction was confirmed by experiments with concatenated heterotetrameric hERG1 channels formed by coassembly of WT and mutant subunits with defined stoichiometry for all three activators (Wu et al., 2014, 2015). Finally, our experimental and modeling findings are inconsistent with a previously proposed model that proposed hERG1 activators do not interact directly with the channel protein but instead modulate the energy barriers that determine channel gating transitions by altering the dipole potential of the cell membrane (Pearlstein et al., 2017).

## Data Availability

The original contributions presented in the study are included in the article/Supplementary Materials, further inquiries can be directed to the corresponding author. The docking coordinates are available on zenodo via the following doi: https://10.5281/zenodo.7594973

## Supplementary material

SUPPLEMENTARY FIGURE S1 The binding site of RPR in the 5VA1 cryo-EM structure **(A)** and a snapshot of the MD simulation **(B)**. Binding residues are represented as spheres, RPR is presented as yellow sticks. Residues represented in pink are known from experiments to prevent to slowing of deactivation and reduced the shift of V_0.5_ of inactivation by RPR. Those in green weakened the effect of deactivation, but did not change the effect of RPR an inactivation. Gray residues are within 5 Å of RPR.

## Abbreviations

C-del, carboxyl terminus deleted; CNBHD, cyclic nucleotide binding homology domain; G-V, conductance-voltage; g/gmax, relative conductance; hERG1, human ether-à-go-go-related gene type 1; I-V, current-voltage; Imax, maximum current; Ipeak, peak outward current; Itail, tail current; Itail-peak, peak tail current; k, slope factor of Boltzmann function; Kv, voltage-gated K+; LQTS, long QT syndrome; N-del, amino terminus deleted; Po, open probability; rERG2, rat ether-à-go-go-related gene type 2; RPR, RPR260243, (3R,4R)-4-[3-(6-methoxyquinolin-4-yl)-3-oxo-propyl]-1-[3-(2,3,5-trifluorophenyl)-prop-2-ynyl]-piperidine-3-carboxylic acid; VSD, voltage sensor domain; Vret, return potential; Vt, test potential; V0.5, half-point (in mV) of Boltzmann function; WT, wild-type; τdeact, time constant of deactivation; MD, molecular dynamics.
