# Supplementary figures and images for: Binding of RPR260243 at the intracellular side of the hERG1 channel pore domain slows closure of the helix bundle crossing gate
Source: Front Mol Biosci. 2023 Feb 23;10:1137368. doi: 10.3389/fmolb.2023.1137368 (PMC9996038; doi:10.3389/fmolb.2023.1137368)

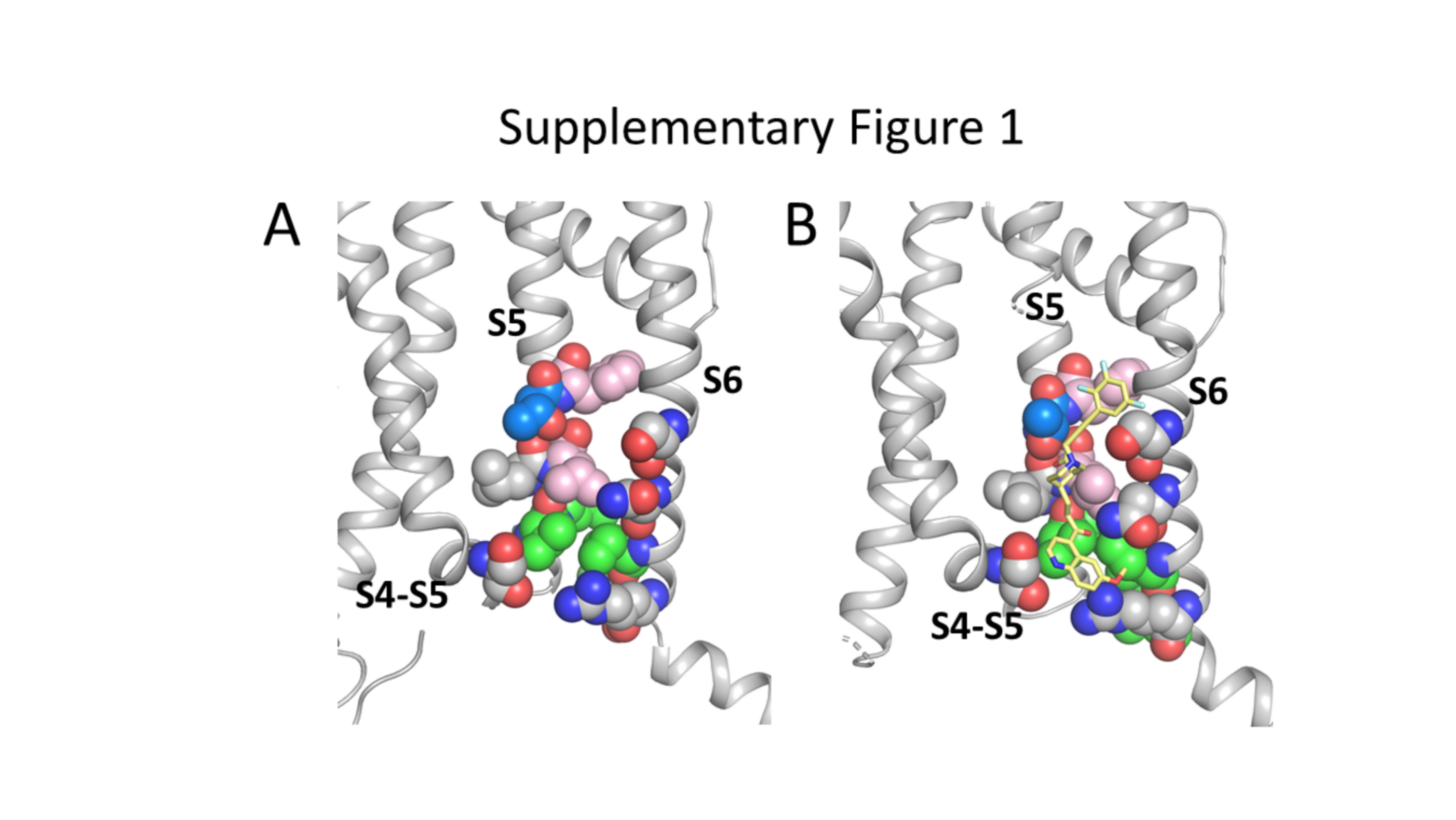

Supplement: Supplementary file 1 [file Image1.TIF]
